# The Antennal Sensory Structures of Female *Anagyrus vladimiri* (Hymenoptera: Encyrtidae)

**DOI:** 10.3390/insects13121138

**Published:** 2022-12-10

**Authors:** Milos Sevarika, Paolo Giannotti, Andrea Lucchi, Roberto Romani

**Affiliations:** 1 Department of Agricultural, Food and Environmental Sciences, University of Perugia, 06121 Perugia, Italy; 2 Department of Agriculture, Food and Environment, University of Pisa, 56124 Pisa, Italy

**Keywords:** SEM, TEM, ultrastructure, parasitoid, sensilla

## Abstract

**Simple Summary:**

Perception of olfactory cues represents a core function of insects. They perceive these signals by small organs, termed as sensilla, mostly located on the antennae. Besides olfactory sensilla, the antennae are also equipped with other types of sensilla involved in the perception of various cues (i.e., contact chemical, mechanical, physical). Through the integration of different stimuli from the environment, insects are able to interact dynamically with the biotic and abiotic components of the ecosystem. The ultrastructural characterization of sensilla allows for a specific function to be assigned to a specific type of sensilla, providing the base for further electrophysiological and ecological studies. In this work, we investigated the antennae of an important biocontrol agent of scale insects, *Anagyrus vladimiri*. The ultrastructural organization of the sensilla was investigated by scanning and transmission electron microscopy. We found eight different types of antennal sensilla, for which we proposed a specific functional hypothesis.

**Abstract:**

The Encyrtidae (Hymenoptera) encompasses a large group of parasitic wasps widely used in biocontrol programs of scale insects (Hemiptera: Coccoidea). *Anagyrus vladimiri* is a solitary parasitoid that attacks and develops on several mealybugs of economic importance. Thus, this species is widely used as a biocontrol agent of *Planococcus* spp. and *Pseudococcus* spp. *A. vladimiri* males and females show sexual dimorphism with regard to the antennal organization, in terms of shape and the development of antennomeres. Ultrastructural investigations of female antennae, carried out with scanning (SEM) and transmission (TEM) electron microscopy, revealed the presence of nine distinct antennomeres. The scape was enlarged and paddle-like, compared to the other antennomeres. The club (the apical antennomere) was mono-segmented and housed the highest number of sensilla. Eight morphologically different types of sensilla were described; sensilla trichoidea I, trichoidea II, chaetica I, chaetica II, grooved peg sensilla, campaniform sensilla, multiporous plate sensilla and multiporous basiconic sensilla. Ultrastructural investigations allowed for us to assign a specific function to each type of sensilla. The most abundant type of sensilla were sensilla trichoidea I and multiporous plate sensilla. We also found two types of sensilla (multiporous basiconic sensilla and sensilla chaetica II) that were present only on the females.

## 1. Introduction

The mealybugs (Hemiptera: Pseudococcidae) are a group of scale insects, recognized as important pests of agricultural and ornamental crops [1]. They cause damage as nymphs and adults by inhibiting plant growth, excreting large quantities of honeydew, causing defoliation, plant damage and ultimately loss in production [2,3]. In addition, they are known as vectors of plant viruses [4,5]. The detection and control of mealybugs are challenging, due to their cryptic behaviour and wax production, which serves as a protective barrier against successful insecticide application [6,7,8,9]. In the framework of integrated pest management (IPM), different biocontrol agents, including predators (e.g., Coccinellidae) and parasitoids (Encyrtidae), are used to suppress the mealybug population. Specifically, endoparasitoids from the genus *Anagyrus* are often included in biocontrol programs [8,10,11,12].

The genus *Anagyrus* encompasses about 350 species of primary endoparasitoid of Pseudococcidae [13]. Within the genus, there are morphologically similar species, such as *Anagyrus pseudococci* (Girault), *A.* sp. near *pseudococci* (Girault), *A. dactylopii* (Howard), *A. kamali* Moursi and *A. kivuensis* Compere. All these species are nominally included in the informally designated *Anagyrus pseudococci* complex, distinguished by the coloration of the female antennal flagellomere 1 (F1) [14]. Because of their morphological similarity, the first two species have been misidentified and are sometimes confused or mistaken for each other by researchers and commercial bio-factories.

Previous research discovered a variation in F1 coloration on female parasitoids between Californian (completely black morphotype) and Argentinian (black/white morphotype) populations. Primarily, it was believed that this minor variation could represent population-specific characteristics. However, further analysis confirmed that the taxon formerly defined as *A. pseudococci* conversely included a different species that was initially classified as *A.* sp. near *pseudococci* (Girault) [15] and then described and named as *Anagyrus vladimiri* Triapitsyn [14].

*A. vladimiri* is a well-known biocontrol agent of *Planococcus* spp. and *Pseudococcus* spp. species. Until now, it has been successfully used to control *Pseudococcus comstocki* (Kuwana), *P. viburni*, *Planococcus ficus* (Signoret) and *P. citri* [15,16].

The success of a biocontrol agent in controlling a pest is partially correlated with its ability to locate a host in an appropriate time window. To do so, insects utilize diverse cues, of which olfactory plays a major role. Some olfactory cues are highly volatile and thus detected through larger distances (pheromones, oviposition induced plant volatiles (OIPV), herbivore induced plant volatiles (HIPV)), contrasting with the less or even non-volatile, like in the case of cuticular hydrocarbons. Volatile cues are detected by olfactory sensilla, while non-volatile by gustatory sensilla [17,18,19]. These, along with other sensilla (mechanoreceptors, thermo-hygroreceptors), are present all over the insect body, but mainly on the antennae, which are recognized as the primary sensory appendages [20].

The morphology and distribution of antennal sensilla in males and females of *A. vladimiri* have been studied with the scanning electron microscopy [21]. Six morphologically different sensilla were found on both sexes: trichoidea, placoidea, basiconica and chaetica type 1, type 3 and type 4, whereas chaetica type 2 sensilla were present only in females, and tridentate scale-shaped-type sensilla were present only in males. Based on sensilla external morphology (the presence or absence of pores), authors have hypothesized a possible olfactory, gustatory or mechanoreceptive function.

In our work, we studied in detail the antennae of *A. vladimiri* with both scanning and transmission electron microscopy, with the aim to define the possible real function of sensilla in the female antennae.

## 2. Materials and Methods

### 2.1. Insects

Commercially mass-reared males and females of *A. vladimiri* were provided by BioPlanet (Cesena, Italy). When emerged, parasitoids were sexed, independently stored in clean glass vials and fed with a tiny drop of water and honey solution (1:1).

### 2.2. Scanning Electron Microscopy (SEM)

Ten *A. vladimiri* adult females and males were used. The insects were anesthetized by freezing and their head capsules, complete with the antennae, were excised from the thorax using fine forceps and placed in a lens cleaner liquid for 24 h. Dehydration was carried out in a graded series of ethanol (from 50% up to 99%) followed by treatment with a critical point dryer Leica EM CPD 030 (Leica Microsystems, Wetzlar, Germany). Then, the specimens were mounted on aluminium stubs with double-sided sticky tape, positioned on dorsal or ventral sides to obtain a total sensillar count for each replicate. The specimens were then sputter-coated with an Edwards sputter coater S150B (BOC Edwards, Burgess Hill, UK) prior to their examination with a Scanning Electron Microscope FEI Quanta 200 (Thermo Fisher Scientific Inc., Hillsboro, OR, USA).

### 2.3. Transmission Electron Microscopy (TEM)

For TEM observations, five females were paralyzed by exposure to cold temperatures (−18 °C) for 60 s, then immediately immersed into a solution of glutaraldehyde and paraformaldehyde 2.5% in 0.1 M cacodylate buffer +5% sucrose, pH 7.2–7.3. Each antenna was detached from its base, single antennomeres were isolated and reduced in size by cutting them in two parts to facilitate fixative penetration and then left at 4 °C for 2 h. The specimens were kept at 4 °C overnight in the same buffer, then they were post-fixed in 1% OsO4 (osmium tetroxide) for 1 h at 4 °C and rinsed in the same buffer. Dehydration, in a graded ethanol series from 60% to 99%, was followed by embedding in Epon-Araldite (Sigma-Aldrich, Dorset, UK) with propylene oxide as a bridging solvent. Thin sections were taken with a diamond knife on a LKB “Nova” ultramicrotome (LKB, Stockholm, Sweden) and mounted on 50 formvar-coated mesh grids. Then, sections on the grids were stained with uranyl acetate (20 min, room temperature) and lead citrate (5 min, room temperature). Finally, the sections were observed with a Philips^®^ EM 208 (Thermo Fisher Scientific Inc., Hillsboro, OR, USA). Digital pictures (1376 × 1032 pixels, 8 bit, uncompressed greyscale TIFF files) were obtained using a high-resolution digital camera MegaViewIII (SIS^®^) (SIS, Muenster, Germany ) connected to the TEM.

## 3. Results

### 3.1. General Description of the Antennae

**Figure 1:**
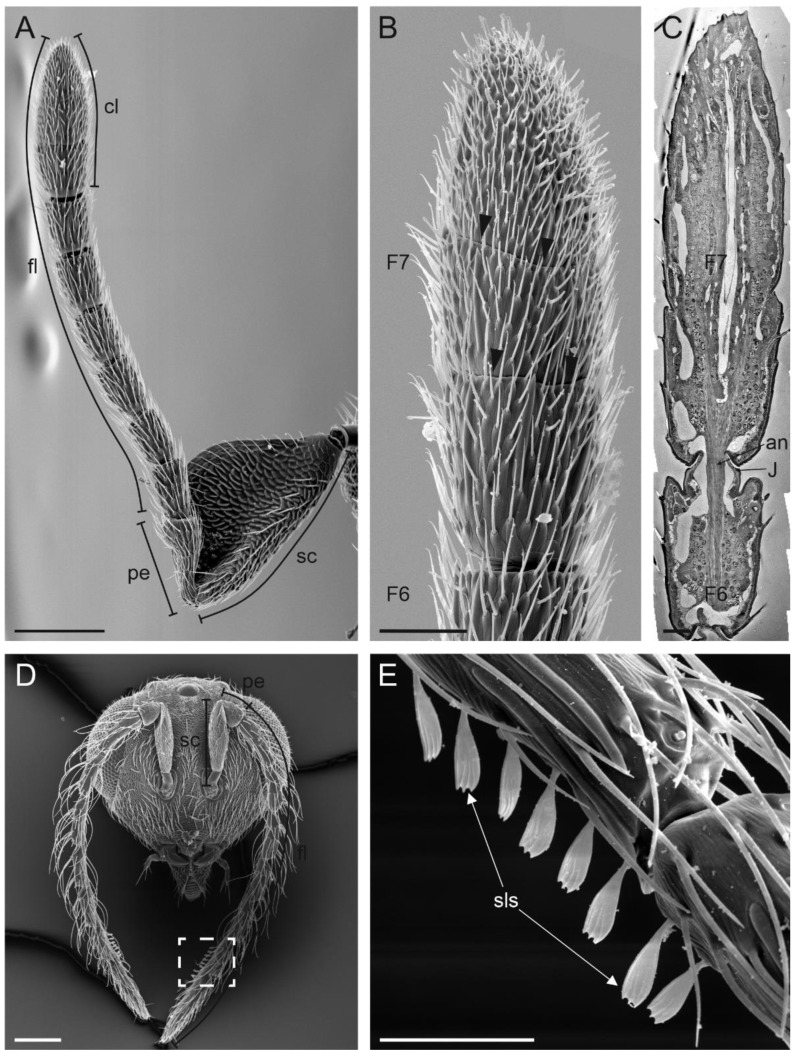
Scanning electron microscopy (SEM) of the *Anagyrus vladimiri* antennae. (**A**) General view of the female antenna showing the paddle-like scape (sc), the pedicel (pe) and the flagellomere made up of seven antennomeres. (**B**) Detail of the 7th flagellomere (F7), forming the apical club (cl). Two transverse furrows are indicated by black arrowheads. (**C**) Transmission electron microscopy image obtained combining 13 adjacent pictures, showing a longitudinal section of the F7 and F6. The dissection plan is positioned at the level of the medial region of the antenna, clearly showing the absence of segments at the F7 outer-grooves level. (**D**) SEM picture showing the male head capsule with the two antennae made of 9 antennomeres. (**E**) Close-up view of the dotted square in (**D**) depicting a detail of the scale-like structures (SLS) occurring ventrally on the last two antennomeres. An: antennal nerve; j: joint. Scale bar: (**A**,**D**): 100 µm; (**B**): 25 µm; (**C**): 10 µm; (**E**): 30 µm.

**Figure 2:**
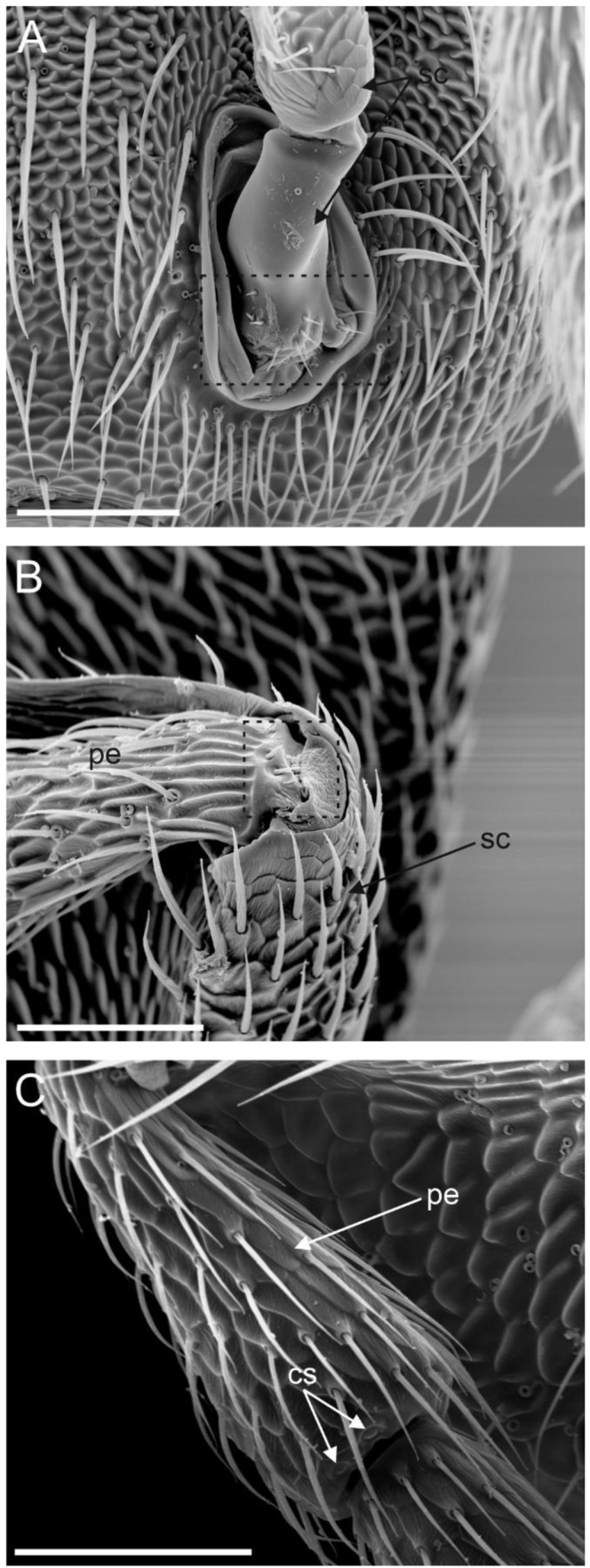
Scanning electron microscopy (SEM) of the *Anagyrus vladimiri* female antennae. (**A**) There is particular focus on the antenna articulation base at the head capsule. The base of the scape is connected with the torulus. The dotted square shows the area where mechanosensory hairs are positioned. (**B**) Detail of the scape (sc)–pedicel (pe) joint; at this level, a few mechanosensory hairs are located near the articulation joint (dotted square). (**C**) Lateral view of the pedicel (pe): two campaniform sensilla (CS) are positioned at the distal end of the antennomere. Scale bar: (**A**,**C**): 50 µm; (**B**): 40 µm.

The antennae of female *A. vladimiri* were the typical antennae found in Hymenoptera, characterised by a geniculate structure and inserted frontally on the head capsule, below the compound eyes and close to the mouthparts. Each antenna was made up of a scape, a pedicel and a flagellum composed of seven flagellomeres (F1–F7); therefore, the whole antenna was made of nine antennomeres (Figure 1A). The scape was inserted in the head capsule through an elliptical socket (Figure 2A). The basal part of the scape was cylindrical and relatively short. At this level, sensilla trichoidea were found arranged close to the head–scape joint (Figure 2A). The most evident region of the scape started in the form of a paddle-like structure, laterally flattened and covered by numerous long hairs (Figure 1A). No pores were found on both sides of any region of the scape. The cuticular surface of the scape presented an elaborate, scale-like sculpture. Occasionally, a few curls of wax originating from the attacked host were observed. The pedicel was attached to the apical corner of the scape, opposite to the rounded edge. Sensilla trichoidea, similar in shape and arrangement to those reported at the head–scape joint, were visible at the scape–pedicel joint (Figure 2B). The pedicel appeared conical in shape, with the diameter increasing from the proximal to the distal part, where sparse campaniform sensilla were found (Figure 2C). Antennomeres ranging from F1 to F6 were cylindrical in shape and similar in size (Figure 1A). The last antennomere (F7) was club-like and resulted from the merging of three originally separated antennomeres (Figure 1A–C). It was the largest antennomere of the antenna—about 180 µm long.

In males, the antenna is made of the same number of antennomeres, but has a different general structure, being longer and slender compared to the female one (Figure 1D). The scape was much smaller and did not show the paddle-like shape. The apical part of the antenna was not clubbed and a single row of scale-like structures was presented on its ventral side (Figure 1E).

### 3.2. Type of Sensilla

**Figure 3:**
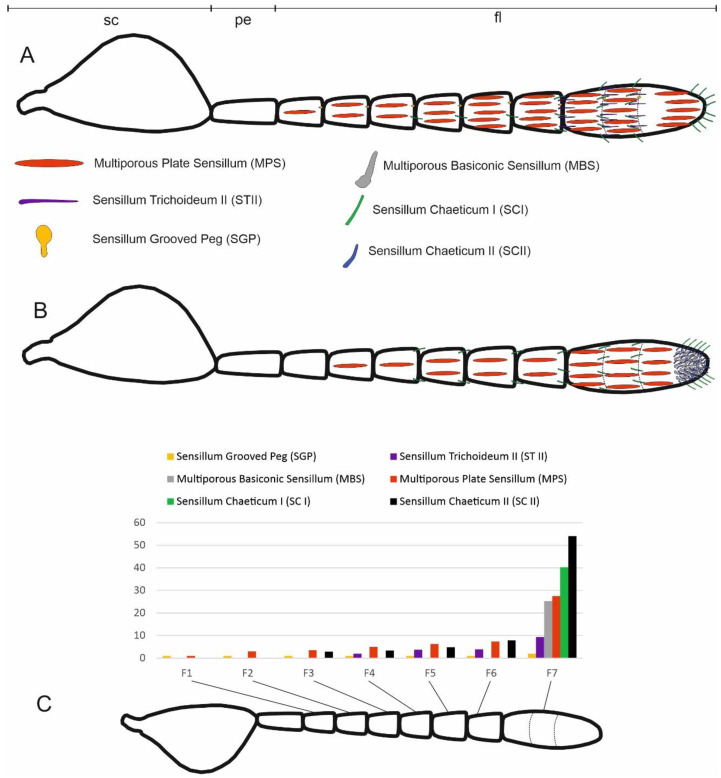
Schematic drawing of *Anagyrus vladimiri* female antennae from dorsal (**A**) and ventral (**B**) views. The different types of sensilla are reported with their relative distribution. Sensilla trichoidea I are omitted. (**C**) Graph showing the relative abundance of the different sensilla types on the different flagellomeres (numbered from F1 to F7). Sc, scape; pe, pedicel; fl, flagellum.

Morphological analysis revealed the presence of eight types of sensilla, which were classified according to their ultrastructural features. A scheme reporting the different sensilla and their distribution along the antennomeres is reported in Figure 3.

### 3.3. Sensilla Trichoidea I (ST I)

**Figure 4:**
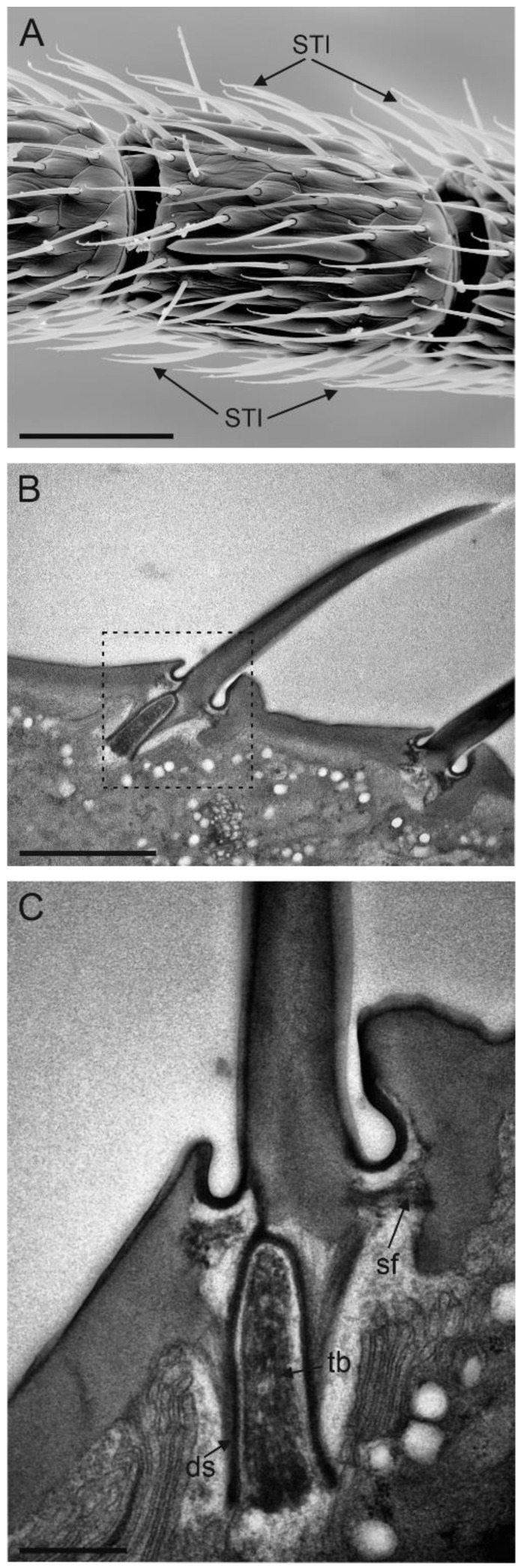
(**A**) SEM picture of flagellomere 5 (F5) of *Anagyrus vladimiri* female antennae showing the distribution of sensilla trichoidea I (ST I). (**B**) TEM picture taken longitudinally through the cuticular shaft and base socket. (**C**) Close-up view of the base socket (dotted square in (**B**) showing the suspension fibres and the single sensory neuron ending in a tubular body (tb) encased by the dendrite sheath (DS). Scale bar: (**A**): 30 µm; (**B**): 2µm; (**C**): 0.5 µm.

Sensilla trichoidea I were the most abundant sensilla on the female antennae. They were present on all antennomeres and arranged in a sequence on both dorsal and ventral sides (Figure 4A). ST I were about 15 µm long and had a bristle-like shape. The external cuticular wall was crossed by longitudinal, shallow furrows throughout its length. The presence of pores was not recorded in ST I (Figure 4B). This characteristic allowed for easy identification from the other subtypes of sensilla trichoidea. At the sensillum base, a distinct socket was present. At this level, TEM cross-sections revealed the presence of a single sensory neuron ending in a tubular body (Figure 4C).

### 3.4. Sensilla Trichoidea II (ST II)

**Figure 5:**
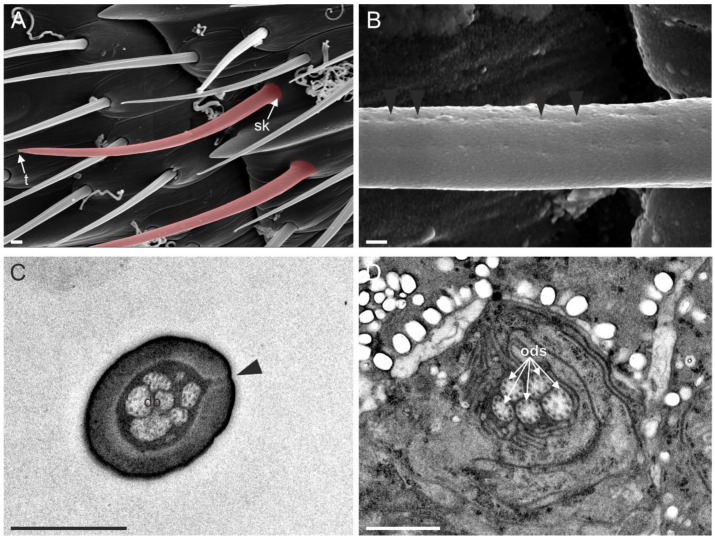
(**A**) SEM picture of the distal area of flagellomere 5 (F5) female antennae in *Anagyrus vladimiri*, two sensilla trichoidea II (ST II) are visible—highlighted with a red mask. The pointed tip (t) and the inflexible socket (sk) are clearly visible. In (**B**), a series of cuticular pores on the surface of ST II can be observed (black arrowheads). (**C**) TEM picture showing a cross-section of an ST II, the section is taken approximately at the medial region of the sensillum. The lumen is occupied by a series of dendritic branches (db), while a pore is visible, connecting the sensillar lumen with the outside (black arrowhead). (**D**) TEM picture of the same sensillum in (**C**) taken below the sensillum base; five outer dendritic segments (ods) belonging to the sensory neurons innervating the sensillum are visible. Scale bar: (**A**,**C**,**D**): 1 µm; (**B**): 0.2 µm.

Sensilla trichoidea II differ from sensilla trichoidea I by being present on the antennae, starting from the sixth antennomere (F4) (Figure 5A). Their number gradually increased from two on F4 to ten on F7. ST II were slightly wider and longer than ST I (about 18 µm in length) and inserted through the antennal wall with an inflexible socket (Figure 5A). Moreover, their external cuticle appeared to be smooth, pierced by tiny pores, mostly organised in parallel lines. The tip was slightly rounded (Figure 5B). A cross-section taken at the apical part of the sensillum revealed the presence of the branched sensory neurons filling the sensillum lumen (Figure 5C). The cuticular wall was thick (~300 nm) and with a few pores (Figure 5C). Sections taken at the basal part of the sensillum showed the presence of five sensory neurons (Figure 5D).

### 3.5. Multiporous Plate Sensilla (MPS)

**Figure 6:**
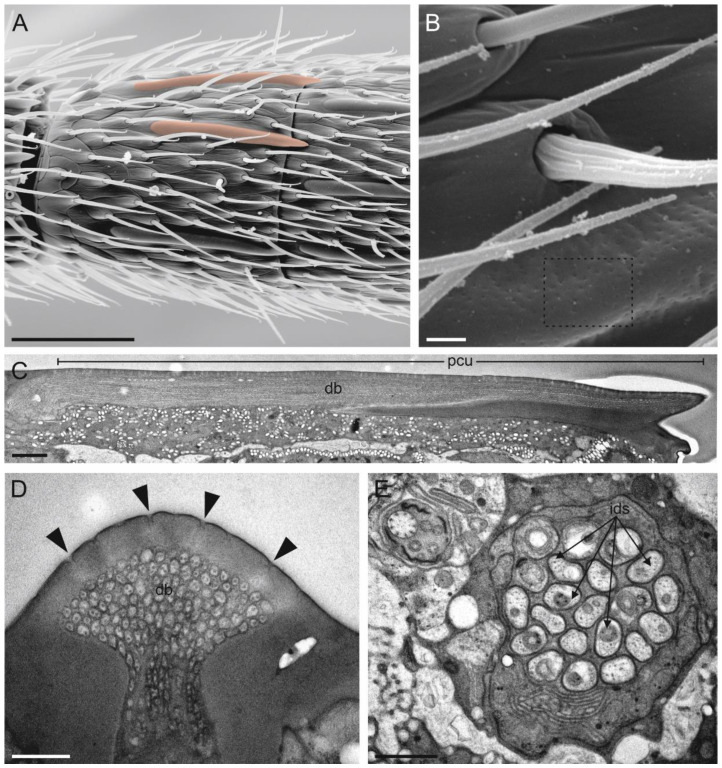
(**A**) SEM picture of the proximal part of F7 in dorsal view, where two multiporous plate sensilla (MPS) can be observed (red mask). (**B**) Detail of the porous cuticle (dotted square). (**C**) TEM image obtained combining 7 adjacent pictures, showing a longitudinal section of an MPS. A large amount of dendrite branches (db) completely fills the space below the porous cuticle (pcu). (**D**) TEM cross-section of an MPS taken approximately at the third distal length form the tip, showing the cuticular pores (black arrowheads) and the dendrite branches (DB). (**E**) TEM cross-section showing the bundle of 20 inner dendritic segments (ids) belonging to the sensory neurons innervating a single MPS. Scale bar: (**A**): 30 µm; (**B**,**E**): 1 µm; (**C**): 2 µm; (**D**): 0.5 µm.

Multiporous plate sensilla were flattened structures that presented a slightly elevated medial part. MPS were longitudinally positioned over the antennomeres and were evenly distributed over the dorsal and ventral side of the antennae (Figure 6A). These sensilla were absent on the scape and the pedicel but present on all flagellomeres. The number of the sensilla gradually increased, being highest at the apical antennomere, where about 28 sensilla were observed. The sensilla were about 42 µm in length. Generally, the sensilla extended over a single antennomere; however, sometimes the sensillum tip protruded over the next antennomere (Figure 6A,C). A close-up view of the external cuticle revealed a sensory cuticle covered by numerous, evenly distributed pores. The transmission electron images revealed the sensillum ultrastructural organization characterized by the multiporous cuticular wall and numerous dendrites (Figure 6C,D). The cross-section images taken at the apical part of the sensillum showed the presence of numerous dendritic branches, while sections taken at the level of the sensory neurons’ inner dendritic segment revealed 20–21 units innervating each MPS (Figure 6E).

### 3.6. Multiporous Basiconic Sensilla (MBS)

**Figure 7:**
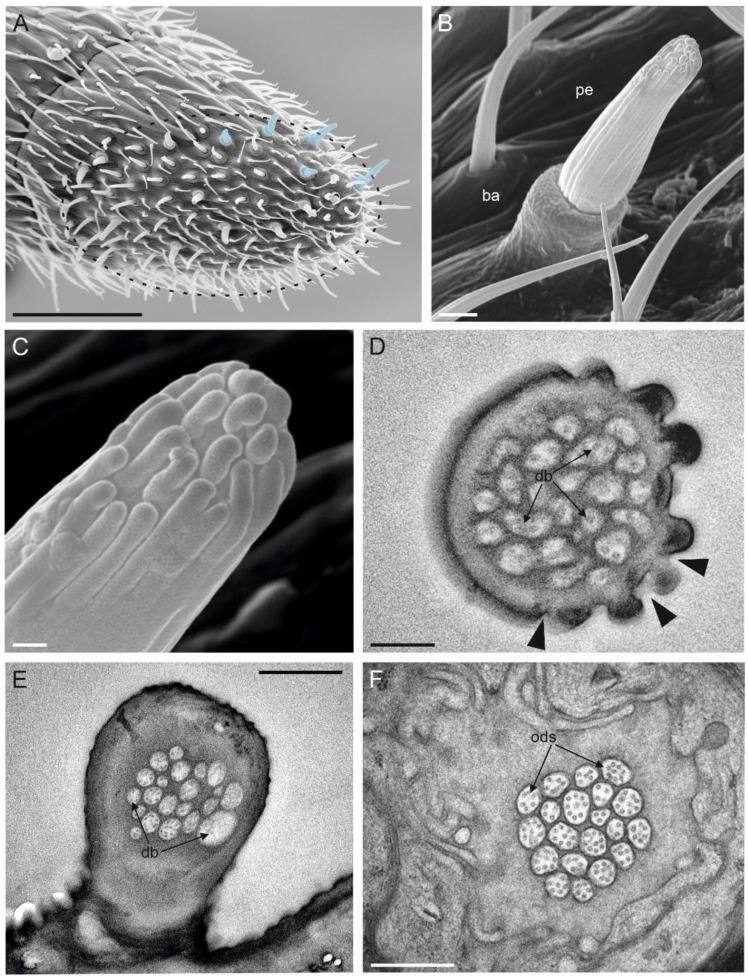
(**A**) Apical part of the female antennal F7 of *Anagyrus vladimiri* in a ventral view. The apical area appears like a flattened sole where several sensilla are inserted. In the blue mask, some of the multiporous basiconic sensilla (MBS) are highlighted. (**B**) Detail of MBS at SEM. The sensillum presents a cylindrical base (BA) that is inflexibly inserted on the antennal wall. A grooved peg (pe) is inserted on top of the cylindrical base. (**C**) Close-up view of the apical region of MBS. The grooves that are present on the cuticular shaft end in elongated finger-like projections. (**D**) TEM picture showing a cross-section of the apical part of MBS. On the right side, it is possible to observe the finger-like projections and the pores (black arrowheads) partly hidden by the same projections. The internal lumen is filled with dendritic branches (db). (**E**) TEM section at the basal region of MBS: the cuticular wall appears thicker and aporous, and the same number of db is visible. (**F**) TEM cross-section through the outer dendritic segments (ODS) of the sensory neurons innervating a single MBS. Scale bar: (**A**): 30 µm; (**B**,**E**): 1 µm; (**C**,**D**): 0.2 µm; (**F**): 0.5 µm.

These sensilla were found exclusively on the apical part of the last flagellomere, inserted on the ventral side (Figure 7A). On average, 25 sensilla were recorded. MBS were characterized by a distinct socket, in the form of a raised cylinder (diameter 2.5 µm) on which a cuticular peg was inflexibly inserted (Figure 7B). The peg cuticle was longitudinally grooved throughout its entire length, which, on average, was 5.5 µm. MBS had a slightly curved tip, on which the grooves running along the peg ended in elongated, finger-like projections (Figure 7C). While cuticular pores were not observed along the sensillum shaft, cross-sections taken at the level of the apical part revealed the presence of several pores defining an apical porous cuticle (Figure 7D). The dendritic projections (~20) of the outer dendritic segments were present within the sensillum lumen (Figure 7E). At the sensillum base, the cuticular wall appeared rather smooth and thick. Each MBS was innervated by ~20 sensory neurons, therefore, they reached the porous cuticle unbranched (Figure 7F).

### 3.7. Sensilla Chaetica I (SC I)

**Figure 8:**
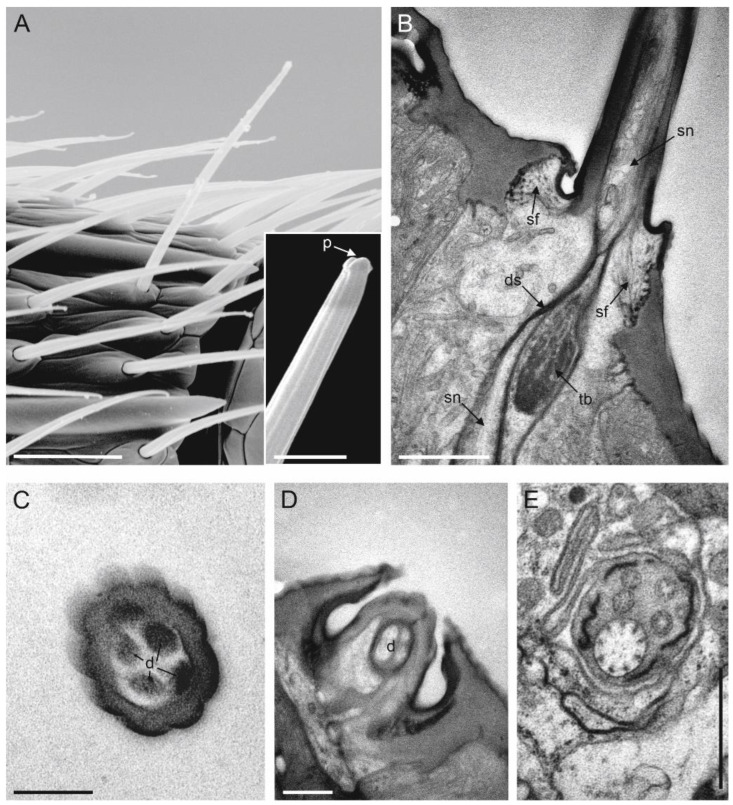
(**A**) Distal region of F6 *Anagyrus vladimiri* female, with evidence of a single Sensillum Chaeticum I (SC I) protruding from the surrounding STI. SC I is characterized by a grooved shaft and a single apical pore (p) (inset in (**A**)). (**B**) TEM cross-section through the socket of a SC I, showing suspension fibres (sf) at the sensillum base and sensory neurons (sn) innervating the sensillum, surrounded by the dendrite sheath (ds). The sensory neurons enter the cuticular shaft of SC I except for the one ending with the tubular body. (**C**–**E**) TEM cross-section taken at different levels of an SC I. In (**C**,**D**), the sections show the dendrites inside the lumen in the medial region of the cuticular shaft (**C**) and close to the base (**D**). In (**E**), the outer dendritic segments are embedded by a discontinuous dendrite sheath. Note the large dendrite belonging to the mechanosensory unit. Scale bar: A: 10 µm; inset in (**A**): 1 µm; (**B**,**E**): 1 µm; (**C**): 0.25 µm; (**D**): 0.5 µm.

These sensilla were characterized by their position and shape. They were typically located at the distal part of each antennomere. The SC I were about 15 µm in length and were inserted on the external cuticle with a 45° angle, which distinguished them from other sensilla (Figure 8A). The external wall presented longitudinal grooves over its entire length; no pores were observed. At the sensillum tip, a single pore could be observed (inset in Figure 8A). SC I could be found from F3 to F7, with a pattern displaying an increase in number from the base to the antennal tip (Figure 3). A total of 73 SC I were recorded per antenna, with most of them (54) located on the club (F7). The ultrastructural TEM investigations revealed the presence of a thick, aporous cuticular wall, with evident ridges as a result of the external grooves (Figure 8C). The sensillum housed five sensory neurons enclosed in a dendritic sheath (Figure 8E). Four sensory neurons developed unbranched dendrites that extended into the sensillar lumen (Figure 8C,D). The fifth sensory neuron ended at the tubular body, located at the sensillum base. Moreover, at this level, suspension fibres connected to the flexible socket could be observed (Figure 8B).

### 3.8. Sensilla Chaetica II (SC II)

**Figure 9:**
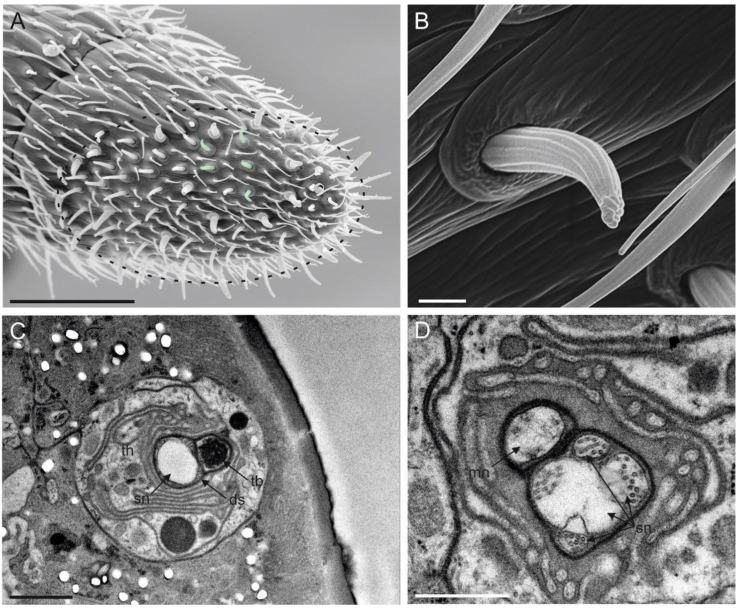
(**A**) Apical part of the female antennal F7 of *Anagyrus vladimiri* in the ventral view: in the green mask, some of the sensilla chaetica II (SC II) are highlighted. (**B**) Detail at the SEM of a SC II. (**C**,**D**) TEM cross-section showing the sensory neurons (SN) associated with a single SC II. In (**C**), a more distal section is reported, where a tubular body (tb) and other sensory neurons (sn) are visible, embedded and separated by the dendrite sheath (ds) and the thecogen cell (th). In (**D**), a more proximal section is shown, reporting the mechanosensory neuron (mn) still separated by the other sn by a distinct ds. Scale bar: (**A**): 30 µm; (**B**,**C**): 1 µm; (**D**): 0.5 µm.

Sensilla chaetica type II were located solely at the apical antennomere; at the same area, MBS were found (Figure 9A). These sensilla showed a cuticular peg characterized by furrows extending over its entire length, ending at the tip and defining an elaborated structure that resembled in part the one reported for MBS (although smaller) (Figure 9B). About 45 sensilla per antenna were recorded, distributed only on the ventral side. The sensilla were inserted on the antennal wall through an evident socket. Although the presence of apical pores was not recorded, we hypothesized the presence of a single pore at the tip. The length on average was 7µm. Ultrastructural investigations with the TEM revealed the presence of five sensory neurons, of which one ended at the tubular body (Figure 9C,D).

### 3.9. Sensilla Grooved Peg (SGP)

**Figure 10:**
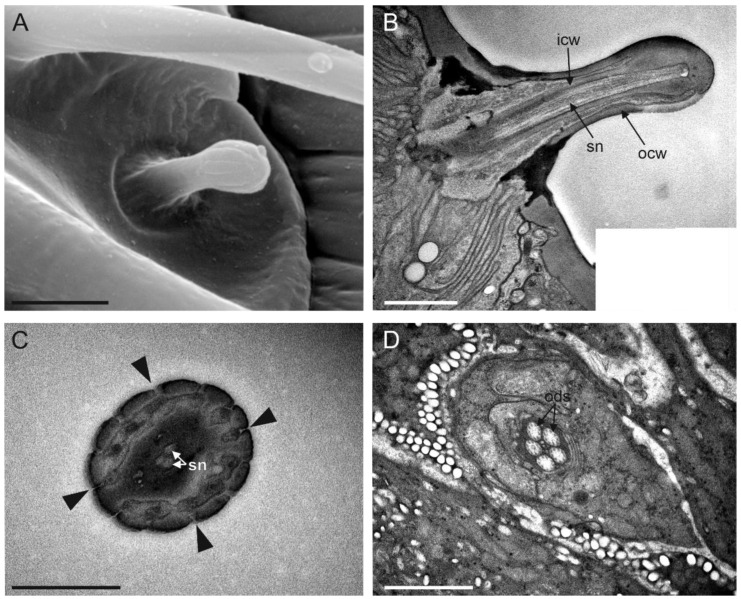
(**A**) A single sensillum grooved peg (SGP) is visible under SEM, located at the distal margin of F6. The sensillum socket appears slightly sunken, while its distal parts are swollen and longitudinally grooved. (**B**) TEM picture obtained from 4 different pictures merged together, showing a longitudinal section of an SGP taken from the tip to the base. Two cuticular walls, the outer (ocw) and inner (icw), are visible, with the icw defining an inner lumen occupied by the sensory neurons (sn). (**C**) TEM cross-section through the sensillum tip sowing the grooves and cuticular pores are somehow hidden between them (black arrowheads). Sn are visible in the central part. (**D**) TEM cross-section taken below the socket level, showing 5 outer dendritic segments (ods) belonging to the sensory neurons associated with a single SGP. Scale bar: (**A**,**D**): 2 µm; (**B**,**C**): 1 µm.

SGP were the least abundant sensilla present on the flagellum. They were distributed on all antennomeres, positioned on the distal antennomere side (Figure 3). We mapped one SGP on each flagellomere, except the last flagellomere, whereby two SGP were present and positioned dorsally. SGP were inserted on the external cuticle through an evident socket, which appeared to be in the form of a slightly depressed ring (Figure 10A). On average, the sensilla were 3 µm long and 1 µm in diameter. The cuticular base was smooth with no pores, while the apical part of the sensillum was rounded with evident grooves (Figure 10A,B). Cross-sections taken at the apical part of the sensillum revealed the presence of a thin multiporous wall, with pores located at the grooves base, and therefore, hidden from the outside (Figure 10C). The longitudinal section through the entire shaft revealed the double-walled nature of SGP, with an outermost and innermost cuticular wall (Figure 10B). This latter gave rise to an inner lumen that was occupied by sensory neurons. While at the apical level, we counted up to three outer dendritic segments—sections taken below the SGP socket revealed a total number of five sensory neurons (Figure 10D).

## 4. Discussion

Encyrtidae is one of the largest families of Chalcidoidea [22]. Their antennae are highly diverse, with flagella appearing from cylindrical to very broad and flat. Although *A. vladimiri* was reported to have a 9-segmented flagellum [14], here we have shown that it is actually composed of 7 segments. This difference is due to the fact that externally, the club appears to be segmented, whereas internally, the last three segments are fused, giving rise to a mono-segmented club. So far, most species belonging to the Encyrtidae were described to present female antennae for which a segmented club was reported [14,23,24,25,26,27,28]; however, in light of what has been observed in *A. vladimiri*, our hypothesis is that, also in other species belonging to the same family, the occurrence of a mono-segmented club is a common trait.

Similar to what has been reported in several other insect species, the distribution pattern of antennal sensory structures in *A. vladimiri* females follows the tendency to have a greater concentration of sensilla in the apical part of the antennae. Based on their ultrastructural organization, the olfactory, gustatory, mechanosensory and thermo-hygroreceptive functions have been hypothesized for the different types of antennal sensilla. In the apical part of the antennae, olfactory and gustatory sensilla are mostly found, while mechanosensory structures are more distributed along the antennal length, with no distinct antennomeres that have a specific abundance. The only exception is the apex of the antennae, which uniquely has two types of sensilla, the multiporous basiconic sensilla and the sensilla chaetica II.

Olfactory sensilla are easily distinguished from mechanoreceptor sensilla, mainly because of their perforated cuticular wall, the presence of internally corresponding pore tubules, and the outer dendritic segments of sensory neurons, characterized by a certain level of branching [20,29]. Therefore, based on our morphological investigations, we hypothesize an olfactory role for multiporous plate sensilla (MPS), sensilla trichoidea II (ST II) and sensilla grooved peg (SGP). The MPS and SGP were widely distributed on the flagellomeres, differently from ST II. As for the sensilla number and distribution, this might depend on the level of specialization in terms of the response to different stimuli. In other words, sensilla that are more widely present and distributed over a larger part of the antennal surface could be associated with the perception of “general odours”, or odours of higher importance (e.g., sex pheromone), while those located in a specific area could be tuned to specific groups of volatile or contact chemical compounds [30].

Multiporous plate sensilla (MPS) are a well-documented and rather frequent type of sensilla in Hymenoptera [31,32,33]. Because of their different external morphology, MPS are often referred to by different names in the literature: porous plates, placoid sensilla, rhinaria, elongate placoid sensilla or multiporous plaque sensilla [32,34,35]. In most families of Hymenoptera Apocrita, MPS are elongated, while in some Vespoidea, Stephanidae and Chrysidoidea, they appear rounded or like small oval plates [33]. In a detailed ultrastructural study of the antennal sensilla of Chalcidoidea, Barlin and Vinson [31] described two types of MPS, which differ in external morphology and cuticular wall thickness. In addition, they studied three species of Encyrtidae, for which they described two types of MPS in females. This contrasts with our results in *A. vladimiri*, where we reported only one type of sensillum, which could be associated with the type I described by Barlin and Vinson. Our observations did not show the presence of a second type of MPS on either the dorsal or ventral side of the antennae. This finding is also confirmed by TEM investigations, which did not reveal the presence of the second type of sensillum, which is characterized by a thick, porous wall. Thus, there seems to be a variation in the presence of MPS types within the different families of Chalcidoidea. No grooves were present around the sensillum, and the tip appeared free of the surrounding antennal cuticle often found in other Chalcidoidea. The internal ultrastructural organization is similar to what has been previously reported. MPS normally have a greater number of associated sensory neurons than those of other sensilla types (frequently more than 20 units). In *Tetrastichus hagenowii* (Ratz) (Eulophidae) and *Torymus warreni* (Cock.) (Torymidae), about 50 sensory neurons have been recorded [31,36], while 25 were recorded in *Dryocosmus kuriphilus* (Cynipidae) [37], 27 in *Itoplectis conquisitor* (Say) (Ichneumonidae) [34], 12–13 in *Coeloide brunneri* Viereck (Braconidae) [38], 37 in *Aphidius smithi* Sharma and Subba Rao (Aphidiidae) [39], and 12–18 in *Apis mellifera* L. (Apidae) [40]. Our finding in *A. vladimiri* of 20 sensory neurons is in line with what has been reported in previous studies. An olfactory function has been proposed for these sensilla, confirmed by electrophysiological studies [41].

Apart from MPS, ST II were also identified as possible olfactory sensilla. This type of sensilla were reported to occur on various insect species [42]. Although the outer cuticular part may show variation among different species (in terms of distribution and abundance of pores), in contrast, its internal organization follows a general pattern typical of an olfactory sensillum. Differently from MPS, ST II were mainly located dorsally on the apical antennomere and at the distal region of the sub-apical antennomere. The ultrastructural organization was similar to the same sensilla in *Paysandisia archon* (Lepidoptera: Castniidae) [43], *Scleroderma guani* (Bethylidae) [44] and *Eupeodes corollae* (Diptera: Syrphidae) [45]. The occurrence of different types of olfactory sensilla is a common trait that can be found in several insects belonging to diverse, unrelated groups (i.e., Auchenorrhycha, [46]; Coleoptera, [47,48]; Lepidoptera, [49,50]).

The less abundant sensilla-type is the grooved peg sensilla (GPS), which are normally found as a singular unit on the distal region of the antennomeres. An exception to this pattern was found in the last flagellomere, where we counted two distinct units. This type of sensilla has been reported for several parasitic wasp groups (i.e., Scelionidae [51,52]; Trichogrammatidae [53]; Mymaridae [54]), for which a role in the detection of volatile stimuli was proposed. Similar sensilla occurred also in specialised, blood-sucking species such as mosquitoes [55] and *Triatoma infestans* Klug [56]. In this last species, a different role for double-walled sensilla, regarding the perception of volatiles, has been proposed, with this sensilla more specifically tuned to more polar and hydrophilic volatiles such as short-chain carboxylic acids. Because of this, a possible role in the perception of green leaf volatiles (being mainly composed by hexenal, hexanol and aliphatic organic acids with a chain length of 3–8 carbons) can be hypothesised for *A. vladimiri*.

From a functional point of view, the presence of different types (or subtypes) of olfactory sensilla has been related to the perception of stimuli of various natures linked to the dynamics of intraspecific interaction or related to the host-selection process. In the case of *A. vladimiri*, olfactory sensilla are most likely involved in the perception of host-derived stimuli directly from the host (i.e., sex pheromones) or from plants attacked by the host (induced volatiles).

In *A. vladimiri*, gustatory sensilla were represented by sensilla chaetica type I (SC I) and II (SC II) and multiporous basiconic sensilla (MBS). In general, from a functional point of view, gustatory sensilla frequently combine mechanoreceptive functions with chemoreceptive gustatory functions [20,29], normally associated with the presence of a single apical pore. Some significant exceptions are reported in Hymenoptera parasitoids, with the occurrence of multiporous gustatory sensilla. This type of sensilla is located exclusively on the apical part of the antennae of females, mainly on the ventral side, which allows for contact between the sensilla and the substrate that the insect explores during host-searching behaviour. Ultrastructurally, they consist of truncated-cone-type cuticular elements that apically have spherical projections that touch each other, but are still flexible, so they can move during antennal contact. The apical part is perforated with pores (however, hidden by the spherical projections), while the cuticular wall of the sensilla is poreless. Internally, the multiporous gustatory sensilla harbour a large number of unbranched sensory neurons. These sensilla have been described in Platygastridae and Scelionidae [57], of which are associated or not with glands. In *Amitus spiniferus* (Platygastridae), multiporous gustatory sensilla have 220 sensory neurons, while in *Trissolcus basalis*, they reach the impressive number of 400 each [52,57]. In *A. vladimiri*, the MBS are innervated by 20–21 unbranched sensory neurons, a value very different from that reported for Playgastridae and Scelionidae. A similar reduction in the number of sensory neurons was found in Trichogrammatidae, in which multiporous gustatory sensilla are innervated by 10 neurons [58]. Such differences in the number of neurons could be associated with the number of multiporous sensilla present. In particular, Platygastridae and Scelionidae have a low number of multiporous gustatory sensilla (whose number does not exceed 10 units per antenna) innervated by a high number of neurons (up to 400 per unit), whereas in *A. vladimiri*, the ventral apical area of females has 25 sensilla, each innervated by 20 neurons. From a functional point of view, in *A vladimiri*, the role of MBS could be to participate in the various stages of the host-selection process by females, a hypothesis in line with what has been reported for other groups of oophagous parasitoids [59].

Sensilla chaetica type I and II shared a typical ultrastructural organization and topography of gustatory sensilla. In fact, they were located on the distal borders of antennomeres (SC I), or at the ventral side of the last antennomere (SC II), which enable them to get in contact with the substrate while the insect is antennating. Both sensilla types are equipped with a mechanosensory neuron that can perceive mechanical stimuli. The number of sensory neurons was the same in both sensilla (four gustatory + one mechanoreceptive neuron). A similar number of sensory neurons was found in other insect groups; *Paysandisia archon* (Lepidoptera: Castniidae) [43]; *Eurygaster maura* (Hemiptera: Scutelleridae) [60]; *Dryocosmus kuriphilus* (Hymenoptera: Cynipidae) [36]; *Harmonia axyridis* (Coleoptera: Coccinelidae) [61]; *Psylliodes chrysocephala* (Coleoptera: Chrysomelidae) [62]. In *A. vladimiri*, SCII are exclusively located at the ventral apical tip of the antennae, therefore, sharing the same topographical region occupied by MBS. A similar situation, whereby sensilla that have the same function (contact chemoreception) are located on the same antennal area, was reported for the fruit fly pupal parasitoid *Trichopria drosophilae* Perkins (Hymenoptera: Diapriidae), where the female is equipped with MGS1 and MGS2; the removal of the apical antennomere impaired the ability of the parasitoid to detect its host [63]. These sensilla are known to respond at various compounds found on the substrate. Electrophysiological recordings carried out in different insect species confirmed that these sensilla act as contact chemoreceptors. For example, electrophysiological recordings on *Trissolcus brochymenae* (Hymenoptera: Platygastridae) showed the response of sensory neurons of sensilla chaetica to various contact stimuli. In addition, a concentration-dependent effect was observed for a few tested compounds, while other compounds had an inhibitory effect on the response of *T. brochymenae* to sugar [64].

On *A. vladimiri* antennae, the major mechanosensory organs were the sensilla trichoidea type I, while sensilla campaniformia and Böhm bristles were located solely at the basal part of the antennae in low numbers. Sensilla trichoidea are a ubiquitous type of sensilla found in different insects. Although the external morphology of sensilla trichoidea is known to differ in length, grooves presence and distribution, their ultrastructural organization is uniform, being made of a sensory neuron ending in a tubular body. For this sensillum, a mechanoreceptive role is hypothesized [29,65,66,67]. Similarly, campaniform sensilla and Böhm bristles are well known for their proprioreceptive mechanosensory function, which can provide information to the insect about the relative position of the antennae during antennation.

Females of *A. vladimiri* were peculiar by having a laterally flattened and enlarged scape—a characteristic commonly reported in Encyrtidae. In other Hymenopterans, the scape is enlarged and sometimes of specific shape, as in the case of the males of *Melittobia australica* Girault [68,69]. These characteristics are usually associated with the specific function that it has on insect biology. For example, the scape of *M. australica* is uniquely shaped in order to clasp the female’s antennae tips during courtship. Moreover, the presence of a pheromone-producing gland located within the scape has been reported [68]. Similarly, in several *Aphelinus* species, the presence of antennal pheromone-producing glands has been recorded, and the crucial role of their products in mediating intraspecific communication was hypothesised [70,71]. In our observations, we did not record any cuticular opening on the *A. vladimiri* female scape, which could indicate the presence of glands. Additionally, apart from some sparse sensilla trichoidea I, no other structures were found, which could suggest a functional role of the enlarged scape. Due to its specific colour pattern, the scape may provide cues for species recognition or could absorb heat due to its dark colour.

In Hymenoptera, sexual dimorphism is often observed in social insects and parasitoids. A similar situation was described in *A. vladimiri,* whereby antennal sensilla play a pivotal role in courtship and mating behaviour [72]. The most prominent differences were the general shape of the antennae (lack of the enlarged scape and the presence of long and elongated antennomeres in males), sensilla number and distribution (the presence of long sensilla trichoidea), and the presence of scales on the ventral side on the male apical flagellomeres. The scales on males of *A. vladimiri* were similar to those described in other Encyrtidae: *Leptomastix dactylopii* Howard, *Rhopus meridionalis* (Ferrière) and *Asitus phragmitis* (Ferrière) [73]. Initially, they were described as specialised male sensory structures. However, detailed ultrastructural organization and behavioural investigations confirmed that they actually represent the release sites of epidermal glands, which produce secretions that act as a contact pheromone, necessary to initiate mating, after which they are named as release and spread structures [73].

## 5. Conclusions

In conclusion, in this study, we reported the ultrastructural organization of the antennal sensilla of female *A. vladimiri*. We showed that the antennae are made of seven antennomeres, with the club being mono-segmented. On the antennae, eight morphologically different sensilla types were recorded, belonging to different functional classes. Among the different types of sensilla, we found two types (MBS and SC II) present only on the female and located in a specific sensory area, for which a role in host recognition was hypothesized. The antennae of males are quite different and have specific structures, for which an involvement in sexual recognition is hypothesized. The present study in fact provides a basis for expanding the study of the functional mechanisms underlying inter- and intraspecific interactions in this important entomophagous species.

## Data Availability

The data that support the findings of this study consist of pictures obtained through SEM and TEM observations. Selected pictures are displayed in the article. All microscopy data are available from the authors upon request.
